# Circulating miRNA expression in asthmatics is age-related and associated with clinical asthma parameters, respiratory function and systemic inflammation

**DOI:** 10.1186/s12931-021-01769-x

**Published:** 2021-06-10

**Authors:** Aleksandra Wardzyńska, Małgorzata Pawełczyk, Joanna Rywaniak, Joanna Makowska, Joanna Jamroz-Brzeska, Marek L. Kowalski

**Affiliations:** 1 Department of Immunology and Allergy, Medical University of Lodz, Poland ul. Pomorska 251, 92-213 Lodz, Poland; 2 Department of Immunology and Infectious Biology, Institute of Microbiology, Biotechnology and Immunology, Faculty of Biology and Environmental Protection, University of Lodz, Lodz, Poland; 3 Department of Rheumatology, Medical University of Lodz, Lodz, Poland

**Keywords:** Asthma, Basic immunology, Elderly, Innate immunity, miRNA, Systemic inflammation

## Abstract

**Background:**

The course of asthma may differ between elderly asthmatics (EA) and non-elderly asthmatics (nEA), which may be partially associated with an age-dependent aberrant immune response. The aim of the study was to determine the influence of serum miRNA expression on asthma characteristics and systemic inflammation markers in EA and nEA.

**Methods:**

Control and severity of asthma, pulmonary function and FeNO were assessed in 28 EA and 31 nEA patients. The control group included 59 elderly and non-elderly healthy individuals. The expression of selected miRNAs in serum was measured with rt-PCR, and proinflammatory cytokine activity was assayed by ELISA or flow cytometry.

**Results:**

No difference in serum miRNA expression was observed between the asthmatics and healthy controls. EA demonstrated lower expression of miRNA-106a and miRNA-126a than nEA (p = 0.003 and p = 0.02) and EC had lower expression of miRNA-146a, -126a, -106a and 19b than nEC (p = 0.001, p = 0.003, p = 0.005 and p < 0.001 respectively). Only nEA demonstrated a relationship between the expression of selected miRNAs and the level of asthma control (assessed with ACT) and with airway inflammation, measured by FeNO level. All patients with asthma demonstrated elevated TNFα, IL-6 and sTNF RI levels compared to controls (p = 0.026, p = 0.03 and p < 0.001 respectively). EA demonstrated a higher TNFα level than EC (p < 0.001), and EA had a higher level of sTNF RI than nEA (p < 0.001). A significant correlation was observed between serum levels of proinflammatory cytokines and selected miRNAs.

**Conclusion:**

Serum miRNA expression was found to correlate with clinical characteristics of asthma and systemic inflammation in an age-dependent fashion, suggesting that miRNA may differentially contribute to asthma pathogenesis in elderly and non-elderly patients.

**Supplementary Information:**

The online version contains supplementary material available at 10.1186/s12931-021-01769-x.

## Introduction

In elderly patients, asthma may be a challenging problem for clinicians as this group display almost all known phenotypes of asthma, which are further modified by physiological ageing and presence of comorbidities [1, 2]. Age-related changes in the respiratory system, such as diminished muscle strength, lung compliance and decreased lung function, may influence the manifestation and severity of asthma symptoms [3]. An important factor that can affect asthma is the progressive impairment of the immune response with age, referred to as *Immunosenecence* [4]. It seems to be associated with an elevated level of proinflammatory cytokines, referred to as systemic inflammation (SI), which may originate from the non-specific activation of innate cells, oxidative stress or persistent viral infections [5, 6]. An increased level of proinflammatory cytokines can be a risk factor for mortality in the elderly population and is found in several inflammatory disorders, including Alzheimer’s disease, diabetes mellitus [7], and in chronic pulmonary obstructive disorders such as COPD [8] and asthma [9–11].

MicroRNAs (miRNAs) are short (18–22 nucleotides), noncoding RNA molecules, that bind to target sequences in mRNA and cause post-translational changes at the epigenetic level [12]. miRNAs can regulate the expression of more than half of mammalian genes [13] and are involved in the control of several physiological processes, including ageing [14]. Dysregulation of miRNAs has also been implicated in the pathophysiology of bronchial asthma [12]. However, the potential role of the serum miRNAs as a biomarker of disease activity and control has not been fully elucidated [15–17].

The aim of the present study was to assess the effect of ageing on serum miRNA expression in asthmatics. It also examines whether age might modify the potential association between miRNAs and clinical features of asthma. Moreover, since miRNAs are involved in the regulation of inflammatory processes, the study also assesses the relationship between miRNAs and selected cytokines known to be important in *Inflammaging* and the pathophysiology of bronchial asthma.

## Materials and methods

### Patients and study outline

The study included 59 patients with asthma diagnosed according to the Global Initiative for Asthma (GINA) 2017 criteria, and 59 control subjects without respiratory disorders. The study subjects were divided into elderly (28 asthmatics and 29 controls aged over 65 years) and non-elderly (31 patients asthmatics and 30 controls aged 30–50) groups. At one appointment, spirometry, impulse oscillometry (IOS), FeNO (Fractional exhaled Nitric Oxide) measurement, and skin prick tests (SPTs) with a panel of inhalant allergens were performed and a blood sample was collected. The Asthma Control Test (ACT) score was recorded and an extended questionnaire, including history of exacerbation, presence of comorbidities was completed.

The clinical and demographical characteristics of asthmatics and controls are presented in Table 1.

**Table 1:** Detailed clinical and demographic characteristics of the study groups

	Asthma, n = 59	Control, n = 59	p-value
Women, n/N (%)	36/59 (61%)	41/59 (69.5%)	ns
age years, mean ± SD	54.5 ± 16.8	52.8 ± 18.3	ns
atopy, n/N (%)	35/59 (59.3%)	24/59 (40.7%)	**0.006**
BMI, median (25–75%)	27.7 (23.9–30.9)	24.8 (22.2–28.4)	**0.02**
BMI > 25, n/N (%)	40/59 (67.8%)	27/59 (45.8%)	**0.01**
FEV1%pred. val., median (25–75%)	93.5 (82.9–105)	110.6 (101.7–113.9)	** < 0.001**
FEV1%/FVC, mean ± SD	69.6 ± 9.3	77.2 ± 8.8	**0.001**
R5Hz %pred. value, median (25–75%)	129.8 (97.1–159.4)	94.8 (84.6–110.7)	** < 0.001**
δR5–R20% pred., median (25–75%)	23.6 (10.7–43.7)	15.4 (8.5–23.1)	**0.006**
δR5–R20 > 20%, n/N (%)	36/58 (62.1%)	19/58 (32.8%)	**0.001**
FeNO ppb, median (25–75%)	21.5 (17.5–28)	20 (17–24.5)	ns
FeNO > 25 ppb n/N (%)	23/57 (40.4%)	13/57 (22.8%)	**0.04**
Number of comorbidities, median (25–75%)	4 (2–7)	1 (0–3)	** < 0.001**
ACT, median (25–75%)	21 (18–25)	–	–

Normally-distributed data are presented as mean ± standard deviation and analysed by Student’s t-test. Variables that were not normally distributed are presented as median and 25–75% percentile (in parentheses) and analysed by the Mann–Whitney test. The χ^2^ test was used for testing relationships between categorical variables. Bold text indicates a statistically significant p-value

*FeNO* fractional exhaled nitric oxide, *ACT* asthma control test, *FEV1% pred.* forced expiratory volume in one second % of predicted value, *FEV1%FVC* forced expiratory volume in one second/forced vital capacity ratio, *R5Hz% pred.* resistance at 5 Hz, % of predicted value, *δR5–20 Hz* difference of resistance at 5 and 20 Hz, *BMI* body mass index

The characteristics of the elderly (EA) and non-elderly asthmatics (nEA) and elderly (EC) and non-elderly controls (nEC) is presented in the Table 2.

**Table 2:** Comparison between elderly and non-elderly asthma patients and controls

	EA, n = 28	nEA, n = 31	EC, n = 29	nEC, n = 30	p-value			
					EA vs. nEA	EA vs. EC	nEA vs. nEC	EC vs. nEC
Women, n/N (%)	17/28 (60.71%)	19/31 (61.29%)	22/29 (75.9%)	19/30 (63.3%)	ns	ns	ns	ns
Age years, mean ± SD	71 ± 5.4	39.5 ± 5.8	70.4 ± 4.8	35.7 ± 5.5	** < 0.001**	ns	ns	** < 0.001**
Atopy, n/N (%)	13/27 (48.15%)	22/25 (88%)	10/29 (34.5%)	14/29 (48.3%)	**0.002**	ns	**0.001**	ns
BMI, median (25–75%)	28.7 (25.3–32.1)	25.4 (20.5–29.3)	26 (24.2–28.5)	24.2 (21.9–27.7)	ns	ns	ns	ns
BMI > 25, n/N (%)	23/28 (82.1%)	17/31 (54.8%)	16/29 (55.2)	11/30 (36.7%)	ns	ns	ns	ns
FEV1%pred. val., median (25–75%)	90.7 (74.8–104.1)	94.7 (86–107.7)	104.4 (99.1–115.9)	110.6 (103.7–113.8)	ns	**0.007**	**0.049**	ns
FEV1%/FVC, mean ± SD	88.4 ± 20.9	94.8 ± 15.1	106.5 ± 14.7	106.9 ± 17.7	ns	**0.002**	** < 0.001**	** < 0.001**
R5Hz %pred. value, median (25–75%)	135 (95.1–173.2)	111.4 (97.1–142.9)	90.4 (72–115.9)	96.8 (88.6–108.8)	ns	**0.003**	**0.046**	ns
δR5–R20% pred., median (25–75%)	32 (16.5–55.9)	20.3 (7.6–34.4)	18.5 (10.1–26.7)	10.8 (1.8–17.7)	ns	ns	ns	ns
δR5–R20 > 20%, n/N (%)	20/27 (74.07%)	16/31 (51.61%)	13/28 (53.6%)	0/30 (0%)	ns	ns	**0.009**	**0.03**
FeNO, ppb, median (25–75%)	22.8 (19.5–28)	20 (14.5–32)	21.8 (18.3–28.5)	18.5 (14.5–22)	ns	ns	ns	ns
FeNO > 25 ppb n/N (%)	11/26 (42.3%)	12/31 (38.7%)	10/28 (35.7%)	3/29 (10.3%)	ns	ns	ns	ns
ACT, median (25–75%)	21 (17–25)	22 (19–25)	–	–	ns	–	–	–
ACT < 20 score, n/N (%)	11/27 (40.74%)	9/31 (29.03%)	–	–	ns	–	–	–
Uncontrolled Asthma according to GINA 2017, n/N (%)	8/27 (29.6%)	4/31 (12.9%)	–	–	ns	–	–	–
Severe asthma according to GINA 2017, n/N (%)	15/28 (53.6%)	16/31 (51.6%)	–	–	ns	–	–	–
Patients with exacerbation/last year, n/N (%)	19/28 (67.9%)	11/31 (35.5%)	–	–	**0.012**	–	–	–
Number of comorbidities, median (25–75%)	7 (4–8.5)	3 (1–3)	3 (2–5)	0 (0–1)	** < 0.001**	**0.02**	** < 0.001**	** < 0.001**

Normally distributed data are presented as mean ± standard deviation, and analysed by Student’s *t*-test. Non-normally distributed data are presented as median and 25–75% percentile (in parentheses). Data are compared between age groups using the Mann–Whitney test or Kruskal–Wallis test followed by Dunn’s Multiple comparison test. χ^2^ test was used for testing relationships between categorical variables

Bold text indicates a statistically significant p-value

*EA* elderly asthmatics, *nEA* non-elderly asthmatics, *EC* elderly controls, *nEC* non-elderly controls, *SI* systemic inflammation, *IOS* impulse oscillometry, *FeNO* fractional exhaled nitric oxide, *SPTs* skin prick tests, *ACT* Asthma Control Test, *FEV1% pred.* forced expiratory volume in one second % of predicted value, *FEV1%FVC* forced expiratory volume in one second/forced vital capacity ratio, *R5Hz% pred.* resistance at 5 Hz, % of predicted value, *δR5–20 Hz* difference of resistance at 5 and 20 Hz, *PBMCs* peripheral blood mononuclear cells, *BMI* body mass index

The study was approved by the Bioethical Committee at the Medical University of Lodz (approval no. RNN/166/14/14/KE). All the study subjects provided their informed consent.

### Skin prick tests

The panel of SPTs (Allergopharma, Reinbek, Germany) included the following inhalant allergens: *Dermatophagoides pteronyssinus*, *Dermatophagoides farinae*, cat, dog, tree mix, grass mix, weed mix, *Alternaria tenuis*, and *Cladosporium herbarum*. A wheal of 3 mm in diameter was regarded as a positive result. Atopy was diagnosed in the event of at least one positive skin test.

### Assessment of respiratory function

Spirometry was performed according to ERS standards [18], using a Vyntus system spirometer (Carefusion, San Diego, California, the United States). Impulse oscillometry (IOS) measurement was performed in triplicate, using an impulse oscillometer Vyntus System (Carefusion, San Diego, California, the United States). The measurements were taken with the patients in a seated position; the patient breathed through a mouthpiece, with a nose clip and the cheeks and mouth supported.

### FeNO measurement

The patients performed single breath maneuvers online according to the ATS/ERS guidelines [19], using the HypAir FeNO (Medisoft, Belgium). The mean value of at least two successful measurements was used for analysis. FeNO measurement was performed before spirometry and IOS.

### Serum miRNA assessment

Three miRNAs, viz*.* miRNA-106a-5p, miRNA-146a-5p and miRNA-19b-3p, were selected for the study based on miRNA profiling in PBMCs isolated from elderly and non-elderly asthma patients and healthy subjects. The profiling results and the outline of the study are briefly presented in the Additional file 1. From the miRNAs whose expression significantly differed in asthmatics and controls in the profiling study, we chose those that had been previously tested in serum and were related to the presence of asthma or its clinical features [15, 20]. Additionally, although not shown as significant in the profiling results, miRNA-126a-5p was also included in the analysis, as many studies have reported it to have an important role in anti-inflammatory responses [20].

The miRNA was isolated from the serum using a miRCURY RNA Isolation Kit-Biofluids (Exiqon, Vedbaek, Denmark) according to the manufacturer's protocol. Equal volumes of RNA were used to prepare complementary DNA (cDNA) with the universal cDNA Synthesis Kit (Exiqon). They were used to perform quantitative PCR (StepOne Plus; Applied Biosystems, Foster City, CA, US) with miR-93 as a reference gene. Serum expression levels of miRNA-106a-5p, miRNA-146a-5p, miRNA-19b-3p and miRNA-126a-5p were evaluated with real-time PCR. miRNA expression is presented as 2^−ΔCT^.

### Measurement of serum cytokines

Proinflammatory cytokines known to be involved in *Inflammaging* [4–8] (TNFα, IL-1β and IL-6, IL-8, IL-12 and sTNF RI) and to be important for the pathogenesis of bronchial asthma were selected for testing. The study also included IL-10, a cytokine with immunoregulatory and antagonistic properties. The serum levels of TNF-α, IL-1β and IL-6 were determined with the use of the commercial Thermo Fisher Scientific Ultrasensitive TNF alpha Human ELISA Kit, High Sensitivity IL-1 beta Human ELISA Kit and High Sensitivity IL-6 Human ELISA Kit. The serum level of soluble TNF RI was determined with the use of the commercial R&D Systems Human TNF RI/TNFRSF1A Quantikine® ELISA Kit. IL-12p70, IL-10 and IL-8 levels in the serum were measured with the BD™ Cytometric Bead Array Human Inflammatory Cytokine Kit. The samples were run on a BD™ LSR Fortessa flow cytometer and the results were analyzed with the BD™ FCAP Array software.

IL-10 was detectable only in 48/118 (40.7%), IL-12p70 in 20/118 (16.9%) and IL-1β in 10/118 (8.5%) of the study participants, and IL-10, IL-12p70 and IL-1β were not included in the analysis.

### Statistical analysis

Normally-distributed data are presented as mean ± standard deviation and analysed by the Student’s *t*-test. Variables that were not normally distributed are presented as median and 25–75% percentile (interquartile range) and analysed by the Mann–Whitney test. Multiple comparisons were analysed using Kruskal–Wallis (KW) test followed by Dunn’s post hoc test. Spearman’s correlation test was used to evaluate correlations between the miRNAs and cytokine expression and clinical/demographic parameters. The χ^2^ test was used to test the relationships between categorical variables.

Univariate and multivariable linear regression was used to investigate the association between each single miRNA, as the dependent variable, and a range of cytokine levels (TNFα, IL-6, IL-8, and sTNF RI) and selected clinical and demographic features: age, FEV1/FVC, sex (women), atopy, elevated FeNO level (> 25 ppb), uncontrolled asthma (ACT score < 20) and obesity (BMI > 25) as independent variables. To be included in the regression models, miRNA expression and cytokine concentration were logarithm (2)-transformed.

The association between miRNA and age in asthmatics and non-asthmatics was studied by univariate and multivariable linear regression with age group*asthma status interaction. In the multivariate model, the level of each of the tested miRNAs was used as the dependent variable, while asthma status, age group (elderly vs. non-elderly), and age group*asthma status interaction were used as independent variables.

The statistical analysis was performed using Statistica 13.1 software (TIBCO Software Inc., USA). p values < 0.05 were accepted as statistically significant.

## Results

### Clinical characteristic of patients

No difference in spirometry or IOS parameters were observed between EA and nEA. Both groups demonstrated a similar level of asthma control and comparable severity according to the GINA criteria. However, the EA participants more frequently reported exacerbation in the last 12 months. Atopy was found more often in nEA than EA.

The comparison between EA and nEA is presented in Table 2.

### Circulating miRNA expression in elderly and non-elderly asthmatics

**Fig. 1:**
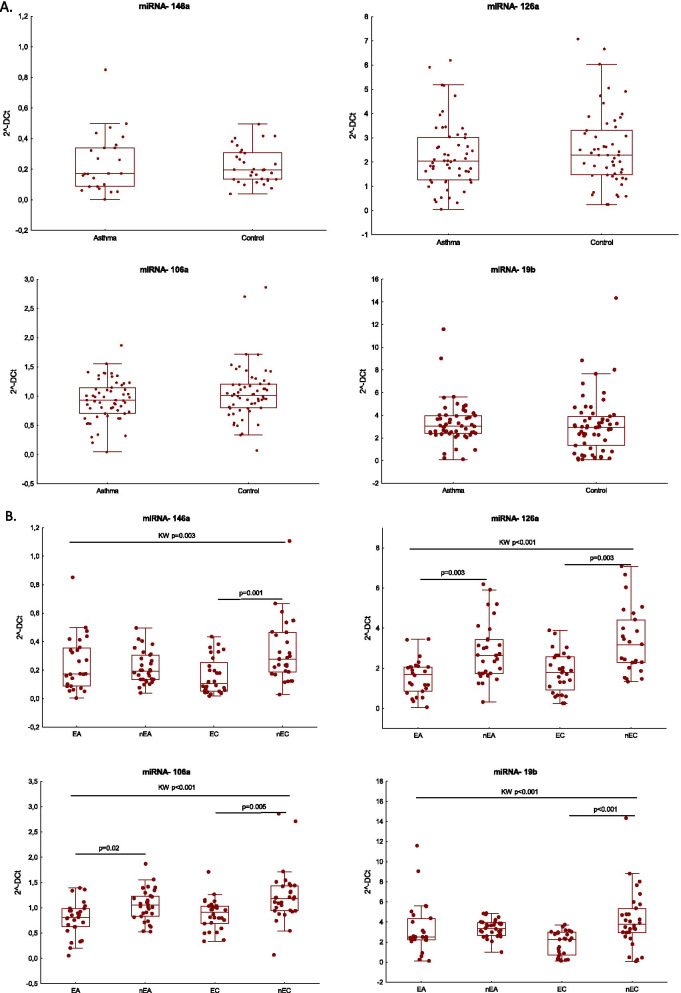
**A** Expression of serum microRNA in asthma patients and controls.** B** Expression of serum miRNA in the age-stratified groups of asthmatics and controls. Data are compared between asthmatics and controls by the Mann–Whitney test and between the age groups by Kruskal–Wallis and Dunn’s Multiple comparison test. The horizontal bar represents the median, box-25–75% percentile, whiskers-range of non-outliers. *EA* elderly asthmatics, *nEA* non-elderly asthmatics, *EC* elderly controls, *nEC* non-elderly controls

No difference in the serum miRNA expression was observed between the asthmatics and healthy controls (Fig. 1A). However, when the age-stratified groups were compared, the elderly asthmatics demonstrated lower serum expression of miRNA -106a and miRNA-126a than nEA, and EC had lower expression of miRNA-146a, -126a, -106a and 19b than nEC (Fig. 1B). In all asthmatics, the serum miRNA -106a and miRNA-126a expression levels correlated with age (r = − 0.33, p = 0.01 and r = − 0.38, p = 0.004, respectively). In EA, a negative correlation was observed between the number of comorbidities and miRNA-106 and -126a (r = − 0.41, p = 0.03 and r = − 0.6, p = 0.001, respectively).

Only the non-elderly asthmatics demonstrated a relationship between the selected miRNAs and the level of asthma control and airway inflammation. ACT was negatively correlated with miRNA-106a (r = − 0.45, p = 0.01) and with miRNA-126a (r = − 0.44, p = 0.01). The FeNO level in the nEA was positively correlated with miRNA-106a (r = 0.62, p < 0.001), miRNA-126a (r = 0.6, p < 0.001) and miRNA-146a (r = 0.41, p = 0.02).

### Proinflammatory cytokine levels in elderly and non-elderly asthma patients

**Fig. 2:**
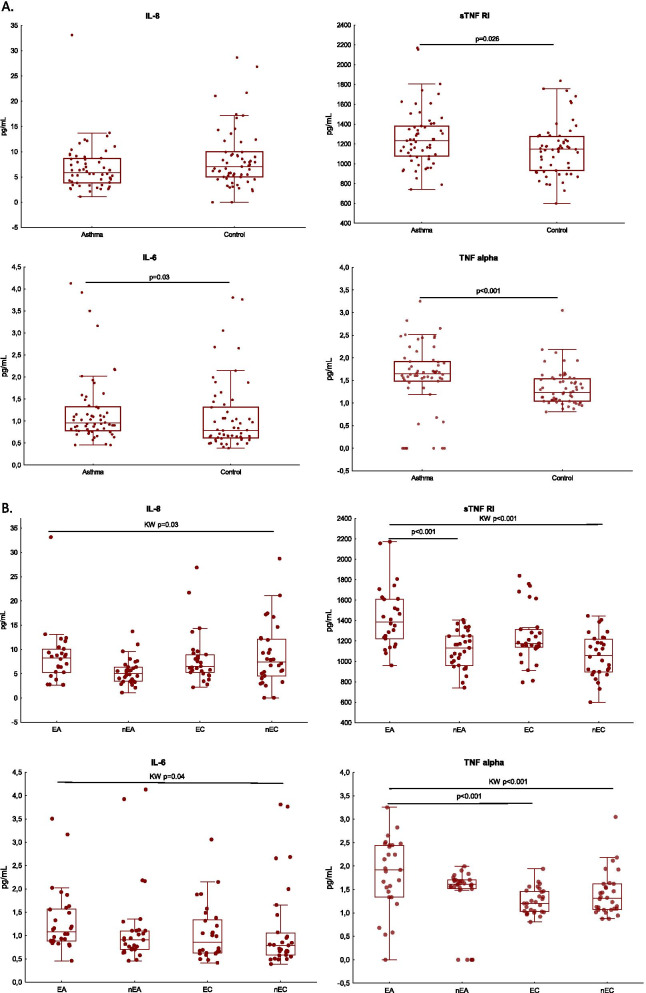
**A** Cytokine levels in asthma patients and in the controls. **B** Cytokine levels in the age-stratified groups of asthmatics and controls. Data are compared between asthma patients and controls with the Mann–Whitney test, and between the age groups with the Kruskal–Wallis and Dunn’s Multiple comparison test. The horizontal bar in represents the median, box-25–75% percentile, whiskers-range of non-outliers. *EA* elderly asthmatics, *nEA* non-elderly asthmatics, *EC* elderly controls, *nEC* non-elderly controls

In all patients with asthma, the TNFα, IL-6 and sTNF RI levels were elevated compared to the control group (Fig. 2A). The EA group demonstrated a higher serum TNFα level than in EC, and elderly asthma patients had higher level of sTNF RI than younger ones (Fig. 2B.).

In the EA sTNF RI was correlated with R5Hz% pred. (r = 0.4, p = 0.04).

A positive correlation was observed between sTNF RI, IL-6 and TNFα levels and BMI (r = 0.48, p = 0.006, r = 0.52, p = 0.003 and r = 0.41, p = 0.02; respectively) in non-elderly asthmatics.

In nEA, serum IL-8 level positively correlated with FEV1 (r = 0.37, p = 0.04) and with R5Hz% pred. (r = 0.4, p = 0.03).

### Serum markers of systemic inflammation correlated with miRNA expression

In all asthma patients, serum sTNF RI levels correlated with miRNA-106a (r = − 0.51, p < 0.001) and miRNA-126a (r = − 0.48, p = 0.004), while in EA, miRNA -126a was negatively correlated with TNF α (r = − 0.54, p = 0.005) and miRNA-146a was positively correlated with IL-8 (r = 0.45, p = 0.03). In the nEA group, a negative correlation was observed between the level of sTNF RI and miRNA-106a (r = − 0.38, p = 0.04), -126a (r = − 0.37, p = 0.045) and -146a (r = − 0.38, p = 0.04)**.**

### Factors associated with miRNA expression

The univariate analysis performed in among all asthmatics found that miRNA-126a and -106a were significantly associated with age and sTNF RI, and that miRNA-126a was associated with TNFα (Table 3). In EA, the univariate analysis revealed a significant relationship between miRNA-126a and sTNF RI; in nEA, miRNA-126a and -106a were associated with elevated FeNO and uncontrolled asthma, and miRNA-106a was associated with sTNF RI level.

**Table 3:** Univariate analysis of association of miRNAs expression with clinical and demographic characteristics in asthmatics and in groups stratified according to age

miRNA	Independent variables	Asthmatics		EA		nEA	
		Beta (95% CI)	p-value	Beta (95% CI)	p-value	Beta (95% CI)	p-value
miRNA-146a	IL-8	0.18 (− 0.1 − 0.45)	0.2	0.26 (− 0.17 to 0.69)	0.22	0.14 (− 0.24 to 0.52)	0.46
	sTNF RI	− 0.18 (− 0.45 to 0.1)	0.2	0 (− 0.44 to 0.44)	0.99	− 0.35 (− 0.7 to 0.01)	0.054
	IL-6	− 0.03 (− 0.3 to 0.25)	0.85	0.17 (− 0.25 to 0.58)	0.41	− 0.24 (− 0.62 to 0.13)	0.19
	TNFα	− 0.16 (− 0.45 to 0.13)	0.27	− 0.17 (− 0.61 to 0.26)	0.42	0.03 (− 0.4 to 0.46)	0.88
	Age	0.01 (− 0.26 to 0.28)	0.96	0.3 (− 0.11 to 0.7)	0.14	0.07 (− 0.31 to 0.45)	0.68
	FEV1%/FVC	0.06 (− 0.21 to 0.34)	0.63	0.02 (− 0.41 to 0.45)	0.92	0.05 (− 0.33 to 0.43)	0.8
	Sex	0.23 (− 0.03 to 0.5)	0.08	0.22 (− 0.19 to 0.63)	0.29	0.29 (− 0.07 to 0.65)	0.11
	FeNO > 25 ppb	0.02 (− 0.25 to 0.3)	0.86	− 0.05 (− 0.49 to 0.39)	0.82	0.15 (− 0.23 to 0.52)	0.43
	Atopy	− 0.13 (− 0.41 to 0.16)	0.38	− 0.13 (− 0.56 to 0.29)	0.53	− 0.23 (− 0.65 to 0.18)	0.26
	BMI > 25	0.01 (− 0.26 to 0.28)	0.96	0.14 (− 0.28 to 0.56)	0.49	− 0.1 (− 0.48 to 0.28)	0.6
	δR5–R20 > 20%	− 0.04 (− 0.31 to 0.23)	0.76	0.18 (− 0.25 to 0.6)	0.4	− 0.27 (− 0.64 to 0.09)	0.14
	ACT < 20	0.07 (− 0.21 to 0.34)	0.63	− 0.05 (− 0.49 to 0.38)	0.8	0.32 (− 0.04 to 0.68)	0.08
miRNA-126a	IL-8	− 0.24 (− 0.51 to 0.03)	0.09	− 0.06 (− 0.5 to 0.38)	0.79	− 0.18 (− 0.57 to 0.21)	0.35
	sTNF RI	− **0.42 (**− **0.68 to ** −**0.17)**	**0.001**	− 0.21 (− 0.64 to 0.22)	0.33	− 0.28 (− 0.65 to 0.09)	0.13
	IL-6	− 0.23 (− 0.5 to 0.04)	0.09	− 0.29 (− 0.69 to 0.12)	0.15	− 0.05 (− 0.45 to 0.34)	0.79
	TNFα	− **0.34 (**− **0.62 to ** − **0.07)**	**0.02**	− **0.44 (**− **0.84 to ** − **0.04)**	**0.03**	0.24 (− 0.18 to 0.67)	0.25
	Age	− **0.42 (**− **0.67 to ** −** 0.17)**	**0.001**	− 0.1 (− 0.52 to 0.32)	0.64	0.06 (− 0.33 to 0.44)	0.77
	FEV1%/FVC	0.19 (− 0.08 to 0.46)	0.16	− 0.04 (− 0.46 to 0.38)	0.84	− 0.06 (− 0.44 to 0.33)	0.76
	Sex	0.06 (− 0.21 to 0.33)	0.67	0.11 (− 0.31 to 0.53)	0.59	− 0.02 (− 0.4 to 0.37)	0.93
	FeNO > 25 ppb	0.04 (− 0.23 to 0.32)	0.76	− 0.2 (− 0.62 to 0.22)	0.34	**0.41 (0.06 to 0.76)**	**0.024**
	Atopy	0.19 (− 0.1 to 0.48)	0.2	0 (− 0.44 to 0.43)	0.98	0.11 (− 0.33 to 0.55)	0.6
	BMI > 25	− 0.18 (− 0.45 to 0.09)	0.18	− 0.21 (− 0.62 to 0.2)	0.3	0.05 (− 0.34 to 0.44)	0.8
	δR5-R20 > 20%	− ** 0.27 (**− **0.53 to ** −** 0.003)**	**0.047**	− 0.19 (− 0.61 to 0.23)	0.36	− 0.25 (− 0.62 to 0.13)	0.19
	ACT < 20	0.02 (− 0.25 to 0.29)	0.89	− 0.24 (− 0.65 to 0.17)	0.23	**0.55 (0.23 to 0.87)**	**0.002**
miRNA-106a	IL-8	− 0.25 (− 0.52 to 0.02)	0.07	− 0.07 (− 0.5 to 0.36)	0.74	− 0.32 (− 0.69 to 0.05)	0.08
	sTNF RI	− **0.46 (**− **0.7 to **−** 0.21)**	** < 0.001**	− 0.27 (− 0.69 to 0.14)	0.19	− **0.39 (**− **0.74 to ** −** 0.04)**	**0.03**
	IL-6	− 0.11 (− 0.38 to 0.16)	0.41	0.07 (− 0.34 to 0.48)	0.73	− 0.19 (− 0.57 to 0.19)	0.32
	TNFα	− 0.11 (− 0.4 to 0.18)	0.45	− 0.09 (− 0.52 to 0.34)	0.68	0.04 (− 0.39 to 0.47)	0.86
	Age	− **0.38 (**− **0.63 to ** −** 0.13)**	**0.003**	− 0.26 (− 0.66 to 0.14)	0.19	0.14 (− 0.24 to 0.51)	0.46
	FEV1%/FVC	0.23 (− 0.04 to 0.49)	0.09	0.09 (− 0.33 to 0.51)	0.65	− 0.03 (− 0.41 to 0.35)	0.87
	Sex	− 0.15 (− 0.41 to 0.12)	0.27	− 0.35 (− 0.74 to 0.04)	0.07	0.17 (− 0.21 to 0.54)	0.91
	FeNO > 25 ppb	0.15 (− 0.12 to 0.42)	0.26	0.06 (− 0.37 to 0.49)	0.77	**0.42 (0.07 to 0.76)**	**0.02**
	Atopy	− 0.01 (− 0.3 to 0.27)	0.93	− 0.21 (− 0.62 to 0.2)	0.3	− 0.09 (− 0.52 to 0.34)	0.67
	BMI > 25	− 0.17 (− 0.44 to 0.09)	0.19	− 0.07 (− 0.48 to 0.34)	0.72	− 0.07 (− 0.45 to 0.31)	0.7
	δR5–R20 > 20%	− 0.17 (− 0.43 to 0.1)	0.22	− 0.13 (− 0.55 to 0.29)	0.53	− 0.01 (− 0.39 to 0.37)	0.95
	ACT < 20	0.22 (− 0.04 to 0.49)	0.09	0.14 (− 0.27 to 0.56)	0.49	**0.67 (0.39 to 0.95)**	** < 0.001**
miRNA-19b	IL-8	0.03 (− 0.25 to 0.31)	0.81	0.18 (− 0.26 to 0.61)	0.4	− 0.04 (− 0.43 to 0.35)	0.84
	sTNF RI	− 0.11 (− 0.39 to 0.16)	0.42	0.11 (− 0.33 to 0.55)	0.6	− 0.25 (− 0.62 to 0.11)	0.17
	IL-6	− 0.14 (− 0.41 to 0.13)	0.3	− 0.17 (− 0.59 to 0.24)	0.41	0.01 (− 0.38 to 0.39)	0.97
	TNFα	0.04 (− 0.25 to 0.34)	0.77	0.08 (− 0.36 to 0.52)	0.72	− 0.13 (− 0.56 to 0.3)	0.54
	Age	− 0.17 (− 0.44 to 0.1)	0.2	0.19 (− 0.23 to 0.6)	0.36	− 0.08 (− 0.46 to 0.3)	0.67
	FEV1%/FVC	0.15 (− 0.11 to 0.42)	0.25	0.03 (− 0.4 to 0.46)	0.87	0.07 (− 0.31 to 0.45)	0.7
	Sex	− 0.01 (− 0.28 to 0.26)	0.94	− 0.03 (− 0.45 to 0.39)	0.89	0 (− 0.38 to 0.38)	0.99
	FeNO > 25 ppb	0.13 (− 0.14 to 0.41)	0.33	0.28 (− 0.14 to 0.7)	0.18	− 0.13 (− 0.51 to 0.24)	0.48
	Atopy	0.06 (− 0.23 to 0.35)	0.68	− 0.08 (− 0.51 to 0.35)	0.7	0.1 (− 0.33 to 0.53)	0.62
	BMI > 25	0.06 (− 0.21 to 0.33)	0.68	0.13 (− 0.29 to 0.55)	0.52	0.25 (− 0.12 to 0.62)	0.17
	δR5–R20 > 20%	0.03 (− 0.24 to 0.31)	0.8	0.15 (− 0.27 to 0.58)	0.46	− 0.01 (− 0.39 to 0.37)	0.97
	ACT < 20	− 0.22 (− 0.49 to 0.05)	0.1	− 0.22 (− 0.64 to 0.2)	0.3	− 0.2 (− 0.57 to 0.17)	0.28

miRNA expression [2^−ΔCt^] and concentration of cytokines [pg/mL] were log-2 transformed before the analysis. Bold text indicates a statistically significant p-value

*EA* elderly asthmatics, *nEA* non-elderly asthmatics, *FeNO* fractional exhaled Nitric Oxide, *ACT* Asthma Control Test, *FEV1%FVC* forced expiratory volume in one second/forced vital capacity ratio, *δR5–20 Hz* difference of resistance at 5 and 20 Hz, *BMI* body mass index

After adjusting for potential confounders in the multivariable regression models, all of the associations listed above turned out to be statistically insignificant (Table 4, Additional file 1).

**Table 4:** Univariate and multivariable analysis of association of miRNAs expression with asthma status, age group and age group*asthma status interaction

miRNA	Variable	Univariate analysis		Multivariable analysis		
		Beta (95% CI)	p-value	Beta (95% CI)	p-value	Model statistics
miRNA-146a	Asthma status	0.01 (− 0.18 to 0.2)	0.92	0.02 (− 0.16 to 0.19)	0.86	**R2 = 0.13, R2 Corr. = 0.11, F(3,111) = 5.5, p = 0.002**
	Age group	− **0.28 (**− **0.46 to **− **0.11)**	**0.002**	− **0.28 (**− ** 0.46 to **−** 0.11)**	**0.002**	
	Age group*asthma status	**Chi2 = 6.27, p(LR) = 0.01**		**0.22 (0.05 to 0.4)**	**0.01**	
miRNA-126a	Asthma status	− 0.09 (− 0.28 to 0.1)	0.35	− 0.12 (− 0.29 to 0.05)	0.17	**R2 = 0.22, R2 Corr. = 0.2, F(3,105) = 9.9, p < 0.001**
	Age group	− **0.45 (**− **0.62 to **− **0.28)**	** < 0.001**	− **0.46 (**− **0.63 to - 0.29)**	** < 0.001**	
	Age group*asthma status	Chi2 = 0.001. p(LR) = 0.98		0 (− 0.17 to 0.17)	0.98	
miRNA-106a	Asthma status	− 0.12 (− 0.3 to 0.06)	0.2	− 0.13 (− 0.3 to 0.05)	0,15	**R2 = 0.12, R2 Corr. = 0.09, F(3,111) = 5, p = 0.003**
	Age group	− **0.31 (**− **0.49 to **− **0.14)**	** < 0.001**	− **0.32 (**− **0.49 to ** −** 0.14)**	** < 0.001**	
	Age group*asthma status	Chi2 = 0.49. p(LR) = 0.49		− 0.06 (− 0.24 to 0.12)	0.49	
miRNA-19b	Asthma status	0.17 (− 0.01 to 0.35)	0.07	0.17 (− 0.01 to 0.35)	0.06	**R2 = 0.13, R2 Corr. = 0.1, F(3,111) = 5.45, p = 0.002**
	Age group	− **0.29 (**− **0.47 to **− **0.12)**	**0.001**	− **0.29 (**− **0.46 to ** −** 0.11)**	**0.002**	
	Age group*asthma status	Chi2 = 2.05, p(LR) = 0.15		0.13 (− 0.05 to 0.3)	0.16	

In the model, each tested miRNA was used as the dependent variable, while the independent variables were asthma status (asthma vs controls), age group (elderly vs. non-elderly), and age group*asthma status interaction. miRNA expression [2^−ΔCt^] was log-2 transformed before the analysis. The bold text indicates a statistically significant p-value

**Fig. 3:**
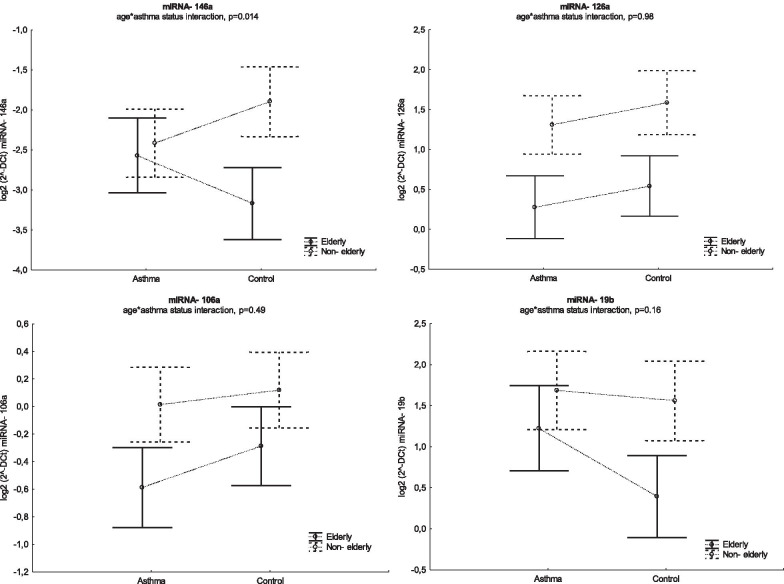
miRNA expression in asthmatics and controls by age group. Circles indicates expected marginal means, whiskers 95% confidence intervals (CI). miRNA expression [2^−ΔCt^] was log-2 transformed before the analysis. The p-value of the age group*asthma status interaction from multivariable regression model with each miRNA as dependent variable is indicated

The age*asthma status interaction was found to be significant in the multivariable regression model with miRNA-146a as a dependent variable (Fig. 3).

Both univariate and multivariable analyses found age to be a significant factor influencing the expression of all miRNAs, regardless of asthma status (Table 4).

## Discussion

The present study examined the relationship of four miRNAs believed to be potentially associated with asthma based on the results of our previous miRNA profiling study, performed on PBMCs collected from elderly and non-elderly asthmatics and age-matched controls. No differences in serum miRNA expression were found between asthmatics and non-asthmatics. However after stratification according to the age, the elderly asthma patients were found to demonstrate lower expression of miRNA-126a and -106a than the younger ones; a similar effect on miRNA-126a, -106a and -19b was observed in the control group. Aging is an important factor influencing expression of miRNAs and most studies report that miRNA expression in serum [21] or in PBMCs [22] is downregulated in elderly subjects. Among all the study participants, i.e. both healthy and asthmatic ones, the elderly individuals demonstrated a significantly lower expression of all miRNAs than the younger ones. A study by Ong et al. [23] examined 285 age-related genes and 27 miRNAs (including downregulated miRNA 146a, -146b and miRNA-142) in lung biopsies obtained from healthy individuals. The authors suggest that in elderly subjects, these miRNAs, e.g. miRNA-146a and -146b, are involved in the regulation of age-related predicted targets responsible for positive regulation of synaptic transmission.

The effect of the aging process is heterogenous for different miRNAs, and very likely depends on the presence of the other factors. The two age groups demonstrated similar tendencies regarding the differences between the levels of miRNA-126a, -106a and -19b between controls and asthmatics. However, elderly patients with asthma had higher level of miRNA-146a as compared to healthy subjects, while the opposite was observed in the younger group. The interaction between age and disease was identified as a significant factor modulating miRNA-146a expression in the multiple regression analysis. According to data from many studies, age-related changes in miRNA levels have also been associated with comorbidities, including coronary heart disease, hypertension, blood pressure and glucose levels [24]. In the present study, only the elderly asthma patients demonstrated a correlation between the expression of miRNA-126a and -106a and the total number of co-morbid diseases, confirming the hypothesis that other disorders with an inflammatory background may also have an impact on age-related miRNA dysregulation. These observations suggest that age and age-related factors (e.g. increased number of comorbidities) may modify the expression of miRNAs to such extent that they cannot serve as reliable asthma biomarkers in elderly individuals.

In this study, the expression of miRNA-106a and -126a was correlated with asthma control in younger patients with asthma; however, no such relationships were observed among the older patients. Other miRNAs have been reported to be differentially expressed in severe asthmatics: including miRNA-221 assayed in airway smooth muscle cells from bronchial biopsies [25] and miR-629-3p, miR-223-3p, and miR-142-3p in sputum cells [26]. A few studies, mostly conducted on younger populations (children and younger adults), indicate that altered expression of selected miRNAs may influence spirometry parameters [27, 28]. A correlation has previously been shown between serum miRNAs and respiratory function in a group of adults with moderate and severe asthma, but only during exacerbation, not on a follow up visit [15].

As miRNAs are involved in regulation of inflammatory processes, the relationship between serum miRNA expression was compared with fractional exhaled NO, considered as a surrogate marker of bronchial eosinophilia and T2 type of inflammation. However, only the non-elderly group demonstrated a relationship between of miRNA-106a, -126a and -146a expression and FeNO level. Similarly, in a recent study by Weidner et al. [29], the expression of several circulating miRNAs, including miRNA-146a and -126, was associated with blood eosinophilia and also with inhaled corticosteroid use. Neutrophilic asthma has also been correlated with a distinct miRNA profile [26], and the expression of plasma miRNA-199a has been found to be elevated in asthmatics with elevated levels of neutrophiles in induced sputum [30].

Several miRNAs have been studied in asthma and allergic disorders and most of them are involved in the regulation of inflammatory processes, and in maintaining the epithelium homeostasis and airway remodeling [12]. Our asthma patients demonstrated significantly elevated proinflammatory cytokine levels (IL-6, TNFα and sTNF RI) compared to healthy controls. In EA and nEA participants, a relationship was also observed between cytokine levels and the presence of impaired respiratory function assessed by oscillometry or spirometry. The role of systemic inflammation in the pathogenesis of asthma has not been fully elucidated. Previously, serum levels of soluble TNF receptors were found to be elevated during asthma attacks in atopic and non-atopic subjects [31], and recent observations of two cohorts of asthmatics found a high IL-6 level to be associated with obesity and a more severe disease phenotype [32]. In SARP cohorts, lL-6 was associated with asthma severity and poor airway function and was also an indicator of non-type 2 asthma [33].

Adipose tissue can also be a major source of proinflammatory cytokines. Canoz et al. [11] showed that ESR, CRP, TNF-$\alpha$, IL-6, and leptin levels in obese asthma patients were higher than in non-obese controls, and TNF-$\alpha$, IL-6, and leptin were elevated in obese as compared to non-obese asthmatics. Our findings indicate that BMI was positively correlated with sTNF RI, TNF-α and IL-6 but only in younger patients with asthma, which suggests that systemic inflammation in elderly is modified by also other factors.

In the present study, miRNA expression was found to be correlated with proinflammatory cytokine level, supporting the hypothesis that miRNA play a role in the regulation of systemic inflammation. A few studies have documented that miRNAs are implicated in Th1–Th2 polarization [34] and the regulation of T2 cytokine expression [35–37]. In PBMCs isolated from asthmatic children, miRNA-126a expression was associated with an imbalance in IL-4/INF gamma production [38]. In a study by Jardim et al. [39], 66 miRNAs, mostly involved in regulating inflammatory pathway genes, were found to be differentially expressed in the epithelial cells of asthmatics as compared to non-asthmatics. miRNA profiling carried out in the cohort of children allowed the identification of three miRNAs (miRNA-146b, -206 and miRNA-720) associated with higher risk of exacerbations in a 1-year follow-up. These miRNAs have been involved in the regulation of inflammation via NF-kβ and GSK3/AKT pathways [40]. Our present findings indicate a negative correlation between miRNA-106a, -126a, -146a and sTNF RI in younger patients with asthma. In the elderly asthma patients, a positive correlation was found between miRNA-146a and IL-8, while miRNA -126a was negatively correlated with TNF-α.

Interestingly, we found no correlations between cytokine concentrations and miRNAs in the control group. The regulation of cytokine levels by miRNAs is a complex process that can be direct and indirect. Results from in vitro or animal studies indicate that the majority of miRNAs serve as negative regulators of cytokine-encoding genes [41]; despite this, miRNAs can be induced by different proinflammatory stimuli, such as IL-1β and TNF [42]. However, in the human model, especially in the presence of disease, defective miRNA regulation may lead to overproduction of cytokines. Such hypothesis was introduced by Pauley et al. [43] who observed a significant increase of miR-146a in PBMCs from rheumatoid arthritis patients, particularly among subjects with active disease; in contrast, no differences in TRAF6 or IRAK1 level, i.e. potential targets of miRNA-146a, were found between patients and controls. In a recent study by Pfeiffer et al. [44], stimulation of endothelial cells resulted in the elevated expression of miR-146a followed by a simultaneous increase in IL-6 and IL-8. The authors attribute these results to the type of stimulus used in the model. Our findings suggest that this complicated mechanism may also be influenced by age [24] and treatment (glucocorticosteroids) [29], known to affect miRNA expression.

Although our study provides interesting observations on the relationship between miRNAs and features of asthma with regard to the aging process, it has some limitations. First, the selection of miRNA was based on profiling performed in PBMCs rather than in serum, and miRNA expression may differ between these two materials. In addition, the role of selected miRNAs as asthma biomarkers related to presence of the disease or clinical characteristics should be investigated in an independent group of patients. Another limitation is related to the sample size and the use of multiple testing, which may lead to potential inflation of type I error rate. Finally, the observations made in this study do not provide an explanation of the mechanism of action of individual miRNAs in asthma pathogenesis.

To conclude, an association between miRNA expression and age was observed in both the control group and in asthma patients. Circulating miRNA expression was associated with selected features of asthma only in younger patients. miRNA correlates with the levels of proinflammatory cytokines in asthmatics, regardless of age.

## Supplementary Information

**Additional file 1. Table 1.** Number of miRNAs with significant changes in expression pattern (on microarrays) in all analyzed groups. miRNAs showing a significant level of regulation has been chosen based on fold change, significance level and array signal intensity. **Table 2**. miRNAs with significant changes in expression pattern in medium exposed PBMCSs in age groups.** Table 3**. miRNAs with significant changes in expression pattern in Rv1b exposed PBMCSs in elderly subjects. **Table 4**. Multivariate association of miRNAs expression with clinical and demographic characteristics in asthmatics and in groups stratified according to age.

## Data Availability

The data that support the findings of this study are available from the corresponding author upon reasonable request.

## Abbreviations

EA: Elderly asthmatics
nEA: Non-elderly asthmatics
EC: Elderly controls
nEC: Non elderly controls
SI: Systemic inflammation
IOS: Impulse oscillometry
FeNO: Fractional exhaled nitric oxide
SPTs: Skin prick tests
ACT: Asthma Control Test
FEV1% pred.: Forced expiratory volume in one second % of predicted value
FEV1%FVC: Forced expiratory volume in one second/forced vital capacity ratio
R5Hz% pred.: Resistance at 5 Hz, % of predicted value
δR5–20 Hz: Difference of resistance at 5 and 20 Hz
PBMCs: Peripheral blood mononuclear cells
BMI: Body mass index
