# Supplementary material for: Circulating miRNA expression in asthmatics is age-related and associated with clinical asthma parameters, respiratory function and systemic inflammation
Source: Respir Res. 2021 Jun 10;22:177. doi: 10.1186/s12931-021-01769-x (PMC8193882; doi:10.1186/s12931-021-01769-x)
Supplement: Supplementary file 1 — Additional file 1. Table 1. Number of miRNAs with significant changes in expression pattern (on microarrays) in all analyzed groups. miRNAs showing a significant level of regulation has been chosen based on fold change, significance level and array signal intensity. Table 2. miRNAs with significant changes in expression pattern in medium exposed PBMCSs in age groups. Table 3. miRNAs with significant changes in expression pattern in Rv1b exposed PBMCSs in elderly subjects. Table 4. Multivariate association of miRNAs expression with clinical and demographic characteristics in asthmatics and in groups stratified according to age. [file 12931_2021_1769_MOESM1_ESM.docx]

***Appendix***

**Methods**

***Isolation of PBMC and PBMC infected with RV1b***

Peripheral venous blood samples (18 mL) were collected from twelve patients, 3 elderly asthmatics (above 65 years of age), 3 younger (aged 30 – 50 years) and 6 age- matched controls into S-Monovette tubes with heparin (Sarstedt, Germany). The entire blood sample was diluted to a final volume of 36 ml with PBS. This suspension was layered on Biocoll separating solution (Biochrom, Berlin, Germany) in a conical tube. After density gradient centrifugation (800xg, 20°C, 20 minutes, without brake), peripheral blood mononuclear cells (PBMCs) were isolated. The layer of PBMCs was collected to a fresh tube and washed twice with sterile PBS (200xg, 10 min., RT) and were counted in hemocytometer. 1x10^6^ cells were seeded in cytometric tubes and were suspended in 0.5 ml of RPMI-1640 medium supplemented with 2% FBS containing the Rhinovirus serotype 1B (RV1b, MOI=1.0) or without virus (negative control). 48h post infection, the cells were centrifuged 300xg for 7 min and cells were suspended in RNAlater solution (Qiagen).

***Isolation of miRNA and miRNA profiling***

RNA was extracted from PBMCs using miRCURY RNA isolation Kit (Exiqon, Denmark). Isolated RNA was sent to Exiqon and all further experiments were conducted at Exiqon Services, Denmark. The quality of the total RNA was verified by an Agilent 2100 Bioanalyzer profile. The hybridization was performed according to the miRCURY LNA™ miRNA Array Instruction manual using a Tecan HS4800™ hybridization station (Tecan, Austria). After hybridization, the microarray slides were scanned and stored in an ozone-free environment. The miRCURY LNA™ miRNA Array slides were scanned using the Agilent G2565BA Microarray Scanner System (Agilent Technologies, Inc., USA) and image analysis was carried out using ImaGene 9.0 software (BioDiscovery, Inc., USA). The quantified signals were background corrected (Normexp with offset value 10) and normalized using quantile normalization method. Sample RNA Quality Control was performed using an Agilent 2100 bioanalyzer. The profile of 2087 miRNAs was assessed 48h post infection in Rv1b infected and non – infected PBMCs.

***Results of miRNA profiling***

Out of 2097 miRNAs on the microarray, 433 were found to be expressed in our samples. miRNA showing significant (p<0.05) and more than a 2-fold regulation (up or down) were included to the analysis. Of these miRNAs, we selected only those that had signal intensity well above the array background (7.5-14.5) (Table 1.).

A subset of 14 miRNAs displayed significant changes in expression between uninfected cells from asthmatic patients and from healthy individuals (Table 1. and Table 2.) The same analysis revealed 20 differently-expressed miRNAs in the elderly subgroup, and only three miRNAs in the young subgroup. In the RV1b-infected PBMCs, a subset of nine miRNAs showed significant changes in expression between elderly asthmatics and healthy subjects (Table 2.). Only one miRNA (miRNA-4732) was induced upon infection with RV1b and this difference was observed only in young, healthy individuals.

Table. 1. Number of miRNAs with significant changes in expression pattern (on microarrays) in all analyzed groups. The miRNAs showing a significant level of regulation were chosen based on fold change, significance level and array signal intensity.

| **Compared groups** | **Number of miRNAs showing a sufficient level of regulation** |
| --- | --- |
| **Asthma (Medium) vs. Healthy (Medium)** | 14 |
| **Elderly asthma (Medium) vs. Elderly healthy (Medium)** | 20 |
| **Young asthma (Medium) vs. Young healthy (Medium)** | 3 |
| **Elderly asthma (RV) vs. Elderly healthy (RV)** | 9 |

Table 2. miRNAs with significant changes in expression pattern in medium-exposed PBMCSs in age groups.

| **asthma vs controls** | **Elderly asthma vs elderly controls** | **Non-elderly asthma vs non-elderly controls** |
| --- | --- | --- |
|  | miRNA-15b |  |
| miRNA-19b-5p | miRNA-19b-5p |  |
|  | miRNA-21 |  |
|  | miRNA-22 |  |
| miRNA-27a-3p | miRNA-27a |  |
| miRNA-29a-3p |  |  |
| miRNA-29b-3p | miRNA-29b |  |
| miRNA-30e-5p |  | miRNA-30e-5p |
|  | miRNA-29c |  |
|  | miRNA-101 |  |
|  | miRNA-106b |  |
| miRNA-142-5p | miRNA-142-5p |  |
| miRNA-223 |  |  |
|  | miRNA-342 |  |
|  | miRNA-351a |  |
|  | miRNA-391 |  |
|  | miRNA-451a |  |
|  | miRNA-491 |  |
| miRNA-1260a | miRNA-1260a |  |
| miRNA-1260b |  | miRNA-1260b |
|  | miRNA-3182 |  |
| miRNA-4286 | miRNA-4286 |  |
| miRNA-4532 | miRNA-4532 | miRNA-4532 |
| miRNA-4695 | miRNA-4695 |  |
| miRNA-4876 |  |  |
| miRNA-5581 | miRNA-5581 |  |

Table 3. miRNAs with significant changes in expression pattern in Rv1b-exposed PBMCSs in elderly subjects.

| **Elderly asthma vs elderly controls** |
| --- |
| hsa-miR-3182 |
| hsa-miR-106a-5p |
| hsa-miR-19b-3p |
| hsa-miR-22-3p |
| hsa-miR-146a-5p |
| hsa-miR-142-5p |
| hsa-miR-4286 |
| hsa-miR-491-3p |
| hsa-miR-451a |

Table 4. Multivariate association of miRNA expression with clinical and demographic characteristics in asthmatics and in groups stratified according to age.

| **miRNA** | **independent variables** | **Asthmatics** | | | **Elderly asthmatics** | | | **Non- elderly asthmatics** | | |
| --- | --- | --- | --- | --- | --- | --- | --- | --- | --- | --- |
|  |  | **Beta (95% CI)** | **p-value** | **Model summary** | **Beta (95% CI)** | **p-value** | **Model summary** | **Beta (95% CI)** | **p-value** | **Model summary** |
| **miRNA- 146a** | **IL-8** | 0.16 (-0.36 - 0.68) | 0.53 | R^2=0.22,  R^2 Corr.=-0.18, F(12,23)=0.55, p=0.86 | -0.1 (-1.3 - 1.09) | 0.84 | R^2=0.47,  R^2 Corr.=-0.6, F(12,6)=0.43, p=0.9 | -0.22 (-1.43 - 0.99) | 0.64 | R^2=0.75,  R^2 Corr.=0.01, F(12,4)=1.02, p=0.55 |
|  | **sTNF RI** | -0.35 (-1.18 - 0.48) | 0.39 |  | -0.31 (-1.76 - 1.13) | 0.61 |  | -0.11 (-1.34 - 1.11) | 0.81 |  |
|  | **IL-6** | 0.09 (-0.5 - 0.68) | 0.75 |  | -0.05 (-1.44 - 1.34) | 0.93 |  | -0.53 (-2.02 - 0.96) | 0.38 |  |
|  | **TNFα** | -0.3 (-0.83 - 0.24) | 0.26 |  | -0.66 (-2.04 - 0.71) | 0.28 |  | 0.79 (-0.67 - 2.25) | 0.21 |  |
|  | **age** | 0.1 (-0.61 - 0.8) | 0.78 |  | 0.75 (-0.71 - 2.22) | 0.25 |  | -0.92 (-2.45 - 0.61) | 0.17 |  |
|  | **FEV1%/FVC** | -0.02 (-0.57 - 0.52) | 0.93 |  | -0.03 (-1.22 - 1.15) | 0.95 |  | -0.76 (-2.57 - 1.06) | 0.31 |  |
|  | **sex** | 0.32 (-0.12 - 0.76) | 0.15 |  | 0.58 (-0.54 - 1.7) | 0.25 |  | 0.51 (-0.53 - 1.55) | 0.24 |  |
|  | **FeNO>25 ppb** | -0.04 (-0.47 - 0.39) | 0.85 |  | 0.21 (-0.73 - 1.16) | 0.6 |  | -0.53 (-2.59 - 1.53) | 0.51 |  |
|  | **atopy** | 0.04 (-0.41 - 0.49) | 0.86 |  | 0.44 (-0.91 - 1.79) | 0.45 |  | -0.22 (-1.47 - 1.03) | 0.65 |  |
|  | **BMI>25** | -0.04 (-0.61 - 0.52) | 0.87 |  | 0.15 (-1.08 - 1.39) | 0.77 |  | -0.8 (-2.9 - 1.29) | 0.35 |  |
|  | **δR5-R20 >20%** | 0.06 (-0.5 - 0.62) | 0.82 |  | 0.13 (-1.16 - 1.43) | 0.81 |  | -0.08 (-1.21 - 1.05) | 0.86 |  |
|  | **ACT<20** | -0.01 (-0.44 - 0.43) | 0.98 |  | -0.09 (-1.44 - 1.26) | 0.88 |  | 0.07 (-1.43 - 1.58) | 0.9 |  |
| **miRNA- 126a** | **IL-8** | -0.11 (-0.5 - 0.29) | 0.58 | R^2=0.57,  R^2 Corr.=0.34, F(12,23)=2.52, p=0.03 | 0.02 (-0.87 - 0.91) | 0.96 | R^2=0.65,  R^2 Corr.=0.04, F(12,7)=1.07, p=0.48 | -0.87 (-2.32 - 0.58) | 0.15 | R^2=0.81, Corr. R^2=0.07, F(12,3)=1.1, p=0.54 |
|  | **sTNF RI** | -0.27 (-0.87 - 0.33) | 0.37 |  | -0.26 (-1.35 - 0.83) | 0.59 |  | -0.25 (-1.68 - 1.19) | 0.62 |  |
|  | **IL-6** | -0.12 (-0.58 - 0.34) | 0.59 |  | 0.21 (-0.93 - 1.36) | 0.67 |  | -0.42 (-2.02 - 1.17) | 0.46 |  |
|  | **TNFα** | -0.39 (-0.77 - 0) | 0.05 |  | -0.8 (-1.78 - 0.18) | 0.09 |  | 0.83 (-1.72 - 3.39) | 0.38 |  |
|  | **age** | -0.21 (-0.71 - 0.3) | 0.41 |  | 0.14 (-0.74 - 1.02) | 0.72 |  | -0.21 (-1.94 - 1.51) | 0.72 |  |
|  | **FEV1%/FVC** | 0.01 (-0.39 - 0.41) | 0.95 |  | 0.16 (-0.74 - 1.06) | 0.68 |  | -0.04 (-2.21 - 2.14) | 0.96 |  |
|  | **sex** | 0.12 (-0.21 - 0.46) | 0.46 |  | 0.38 (-0.5 - 1.25) | 0.34 |  | 0.2 (-1.06 - 1.46) | 0.65 |  |
|  | **FeNO>25 ppb** | 0.1 (-0.23 - 0.43) | 0.55 |  | 0 (-0.69 - 0.69) | 1 |  | -0.69 (-4.23 - 2.86) | 0.58 |  |
|  | **atopy** | 0.2 (-0.14 - 0.53) | 0.23 |  | 0.07 (-0.84 - 0.98) | 0.86 |  | -0.29 (-1.69 - 1.11) | 0.56 |  |
|  | **BMI>25** | 0.15 (-0.27 - 0.57) | 0.47 |  | 0.42 (-0.58 - 1.41) | 0.36 |  | 0.26 (-2.11 - 2.62) | 0.75 |  |
|  | **δR5-R20 >20%** | -0.11 (-0.52 - 0.31) | 0.6 |  | -0.32 (-1.32 - 0.68) | 0.47 |  | -0.87 (-2.19 - 0.44) | 0.12 |  |
|  | **ACT<20** | -0.11 (-0.46 - 0.23) | 0.5 |  | -0.5 (-1.37 - 0.37) | 0.22 |  | 0.88 (-1.54 - 3.3) | 0.33 |  |
| **miRNA- 106a** | **IL-8** | -0.07 (-0.51 - 0.38) | 0.75 | R^2=0.43,  R^2 Corr.=0.14, F(12,24)=1.5, p=0.19 | 0.37 (-0.62 - 1.36) | 0.41 | R^2=0.56,  R^2 Corr.=-0.2, F(12,4)=0.74, p=0.69 | -0.1 (-1.11 - 0.9) | 0.79 | R^2=0.83, Corr. R^2=0.32, F(12,4)=1.64, p=0.34 |
|  | **sTNF RI** | -0.55 (-1.24 - 0.14) | 0.11 |  | -0.32 (-1.49 - 0.85) | 0.54 |  | -0.41 (-1.42 - 0.6) | 0.32 |  |
|  | **IL-6** | 0.28 (-0.2 - 0.76) | 0.25 |  | 0.59 (-0.49 - 1.67) | 0.24 |  | 0.65 (-0.59 - 1.89) | 0.22 |  |
|  | **TNFα** | -0.24 (-0.69 - 0.2) | 0.27 |  | -0.09 (-1.2 - 1.03) | 0.86 |  | 0.04 (-1.17 - 1.25) | 0.93 |  |
|  | **age** | -0.1 (-0.7 - 0.51) | 0.74 |  | -0.63 (-1.77 - 0.52) | 0.23 |  | 0 (-1.27 - 1.27) | 1 |  |
|  | **FEV1%/FVC** | 0.09 (-0.36 - 0.55) | 0.67 |  | 0.24 (-0.73 - 1.21) | 0.58 |  | 0.2 (-1.3 - 1.71) | 0.73 |  |
|  | **sex** | -0.13 (-0.5 - 0.25) | 0.49 |  | -0.47 (-1.39 - 0.45) | 0.26 |  | 0.54 (-0.32 - 1.4) | 0.16 |  |
|  | **FeNO>25 ppb** | 0.16 (-0.2 - 0.52) | 0.36 |  | -0.25 (-1.03 - 0.53) | 0.47 |  | 0.3 (-1.41 - 2) | 0.66 |  |
|  | **atopy** | -0.28 (-0.64 - 0.09) | 0.13 |  | -0.67 (-1.69 - 0.35) | 0.16 |  | -0.15 (-1.18 - 0.89) | 0.71 |  |
|  | **BMI>25** | 0.06 (-0.42 - 0.54) | 0.78 |  | -0.01 (-1.01 - 1) | 0.99 |  | 0.02 (-1.72 - 1.75) | 0.98 |  |
|  | **δR5-R20 >20%** | -0.22 (-0.68 - 0.24) | 0.34 |  | -0.34 (-1.37 - 0.69) | 0.46 |  | -0.18 (-1.12 - 0.76) | 0.62 |  |
|  | **ACT<20** | 0.27 (-0.09 - 0.62) | 0.14 |  | -0.07 (-1.17 - 1.03) | 0.89 |  | 0.65 (-0.6 - 1.89) | 0.22 |  |
| **miRNA- 19b** | **IL-8** | 0.31 (-0.17 - 0.79) | 0.2 | R^2=0.33,  Corr R^2=-0.02, F(12,23)=0.93, p=0.53 | 0.49 (-0.56 - 1.55) | 0.3 | R^2=0.58,  R^2 Corr.=-0.26, F(12,6)=0.69, p=0.72 | 0.13 (-1.19 - 1.45) | 0.8 | R^2=0.71, Corr. R^2=-0.17, F(12,4), p=0.65 |
|  | **sTNF RI** | 0.18 (-0.59 - 0.95) | 0.63 |  | 0.14 (-1.13 - 1.42) | 0.79 |  | -0.18 (-1.52 - 1.15) | 0.72 |  |
|  | **IL-6** | -0.25 (-0.8 - 0.3) | 0.35 |  | -0.42 (-1.65 - 0.81) | 0.43 |  | -0.15 (-1.77 - 1.48) | 0.81 |  |
|  | **TNFα** | -0.02 (-0.52 - 0.48) | 0.94 |  | 0.07 (-1.15 - 1.29) | 0.89 |  | -0.01 (-1.61 - 1.58) | 0.98 |  |
|  | **age** | -0.45 (-1.11 - 0.2) | 0.17 |  | 0.43 (-0.87 - 1.72) | 0.45 |  | -0.71 (-2.37 - 0.96) | 0.3 |  |
|  | **FEV1%/FVC** | 0.13 (-0.38 - 0.63) | 0.6 |  | 0.57 (-0.48 - 1.62) | 0.24 |  | -0.06 (-2.04 - 1.91) | 0.93 |  |
|  | **sex** | -0.04 (-0.45 - 0.37) | 0.84 |  | -0.19 (-1.18 - 0.8) | 0.66 |  | 0.32 (-0.81 - 1.45) | 0.48 |  |
|  | **FeNO>25 ppb** | 0.22 (-0.18 - 0.62) | 0.28 |  | 0.26 (-0.57 - 1.1) | 0.47 |  | 0.45 (-1.8 - 2.69) | 0.61 |  |
|  | **atopy** | 0.03 (-0.38 - 0.45) | 0.86 |  | 0.36 (-0.83 - 1.55) | 0.49 |  | 0.07 (-1.29 - 1.43) | 0.89 |  |
|  | **BMI>25** | 0.06 (-0.47 - 0.59) | 0.81 |  | 0.08 (-1.01 - 1.17) | 0.86 |  | -0.09 (-2.37 - 2.2) | 0.92 |  |
|  | **δR5-R20 >20%** | 0.14 (-0.38 - 0.65) | 0.59 |  | 0.26 (-0.89 - 1.4) | 0.61 |  | -0.16 (-1.39 - 1.07) | 0.74 |  |
|  | **ACT<20** | -0.34 (-0.75 - 0.06) | 0.09 |  | -0.49 (-1.69 - 0.7) | 0.35 |  | -0.64 (-2.28 - 1) | 0.34 |  |

In the multivariate model, the dependent variable was each of the miRNA tested, while the independent variables were cytokine levels (TNFα, IL-6, IL-8, and sTNF RI) and selected clinical and demographic features: age, FEV1/FVC, sex (women), atopy, elevated FeNO level (>25 ppb), uncontrolled asthma (ACT score<20) and obesity (BMI>25). miRNA expression [2^-ΔCt^] and concentration of cytokines [pg/mL] were log-2 transformed before the analysis.

EA- elderly asthmatics, nEA- non-elderly asthmatics, FeNO- fractional exhaled Nitric Oxide, ACT- Asthma Control Test, FEV1%FVC- forced expiratory volume in one second/forced vital capacity ratio, δR5-20Hz- difference of resistance at 5 and 20 Hz, BMI- body mass index
